# Improved and customized dengue serodiagnostics through combined NS1/IgM testing and novel dual-cut-off IgG ELISA

**DOI:** 10.1371/journal.pntd.0014295

**Published:** 2026-04-27

**Authors:** Sandra Saschenbrecker, Nadja Muigg, Oliver Klemens, Julia M. Klemens

**Affiliations:** Institute for Experimental Immunology, Affiliated to EUROIMMUN Medizinische Labordiagnostika AG, Lübeck, Germany; Colorado State University, UNITED STATES OF AMERICA

## Abstract

**Background:**

Accurate diagnostics of dengue virus (DENV) infection are essential for patient management, outbreak control, and vaccine implementation. Serological testing plays a key role, especially when molecular assays are unavailable or viremia subsides; yet, cross-reactivity with other flaviviruses remains a challenge. This study examined the diagnostic accuracy of four Euroimmun ELISAs, including a newly developed dual-cut-off IgG ELISA.

**Methods:**

The Dengue Virus NS1 ELISA, Anti-Dengue Virus Type 1–4 ELISA (IgM), Anti-Dengue Virus Type 1–4 ELISA (IgG; native antigen/gE-based), and the novel Anti-Dengue Virus NS1 ELISA 2.0 (IgG; recombinant NS1-based, with an alternative higher cut-off for flavivirus-endemic regions) were analyzed. Sensitivity was determined using sera from 22 Vietnamese patients with RT-PCR-confirmed DENV infection, collected during acute (t1, 1–6 dpo), early convalescent (t2, 4–9 dpo), and late convalescent (t3, 13–19 dpo) phases. Specificity was assessed with samples from 500 healthy German blood donors (HBD) and 40 patients each with West Nile virus (WNV) or Zika virus (ZIKV) infection.

**Results:**

Sensitivities were 90.5%/70.0%/0% (t1/t2/t3) for NS1, 33.3%/85.0%/77.3% for IgM, 66.7%/100%/100% for IgG, and 33.3%/65.0%/100% vs. 19.1%/50.0%/100% for IgG 2.0 (standard vs. alternative cut-off). Combined NS1/IgM testing achieved 100% sensitivity in single acute-phase samples. Combined IgM and IgG 2.0 testing confirmed recent infection by IgM/IgG seroconversion or ≥4-fold IgG increase in 100% of paired samples. Overall specificity was 85.7% (HBD/WNV/ZIKV: 95.0%/50.0%/5.0%) for IgG, compared to 95.7% (98.2%/95.0%/65.0%) and 99.5% (99.8%/97.5%/97.5%) for IgG 2.0 using standard and alternative cut-offs, respectively.

**Conclusions:**

Euroimmun ELISAs support customized, highly accurate and versatile diagnostic strategies applicable to various dengue testing contexts. Combining NS1 and IgM ELISAs may offer a practical alternative to molecular assays during acute infection. The native antigen/gE-based IgG ELISA enables early sensitive IgG detection, although with limited specificity. With minimal cross-reactivity, the NS1-based dual-cut-off ELISA 2.0 (IgG) reliably captures DENV-specific IgG dynamics and enhances differentiation from other flaviviruses, which could provide an advantage in the use for convalescent-phase diagnostics, epidemiological surveillance, and pre-vaccination screening.

## Author summary

Dengue is one of the most widespread mosquito-borne viral diseases worldwide, and timely, accurate case confirmation is essential for patient care and outbreak control. However, diagnosis remains challenging because early symptoms are often non-specific. Furthermore, dynamic viral and serological markers over the course of infection and potential cross-reactivity with related flaviviruses can affect serodiagnostics. In this study, we evaluated a set of four enzyme-linked immunosorbent assays (ELISAs) targeting dengue virus antigen (NS1) and antibodies (IgM and IgG). We demonstrate that combining NS1 and IgM testing enables reliable identification of acute dengue infection from a single blood sample, which is particularly useful in settings where molecular methods are not available. We also assessed a new NS1-based IgG ELISA with an optional alternative cut-off value. Compared with an IgG ELISA based on native antigen/gE, the use of this assay reduces cross-reactivity in individuals exposed to Zika or West Nile virus. The alternative cut-off additionally allows adaptation of testing to different epidemiological settings. Overall, our findings illustrate how tailored serological strategies can improve dengue diagnosis and support more accurate determination of dengue immune status in epidemiological surveillance.

## Introduction

Dengue virus (DENV) belongs to the genus *Flavivirus* (family *Flaviviridae*), which also includes other arthropod-borne human pathogens such as Zika virus (ZIKV), West Nile virus (WNV), Yellow fever virus (YFV), Japanese encephalitis virus (JEV), and tick-borne encephalitis virus (TBEV) [1]. DENV is among the fastest-spreading mosquito-transmitted viruses, with about 4 billion people living in regions at risk of dengue. Each year, an estimated 400 million infections occur globally, including 96 million symptomatic cases [2–5]. Due to substantial morbidity and mortality, dengue poses a major public health challenge [6]. Its incidence has increased dramatically in recent decades, driven by climate change, expanding mosquito habitats, limited healthcare infrastructure, and rapid urbanization [5,7]. Endemic in South-East Asia, the Americas, the Western Pacific, Africa, and the Eastern Mediterranean, DENV is now emerging in previously dengue-naïve regions, including parts of Europe and South America [3,8,9].

Approximately 75% of DENV infections are asymptomatic [10]. Symptomatic cases range from mild to moderate illness and can rapidly progress to severe dengue, characterized by plasma leakage, shock, internal hemorrhage, and organ failure. Severe dengue is a medical emergency and can be fatal without prompt treatment [11–13]. Risk factors for severe disease include prior infection with a heterologous DENV serotype, viral genotype, prior flavivirus exposure, timing between infections, and host factors such as genetics, age, sex, and comorbidities [12,14].

Vaccines such as Dengvaxia (Sanofi Pasteur) and Qdenga (TAK-003, Takeda) are approved and indicated for preventing dengue; however, Dengvaxia is limited to individuals with a test-confirmed previous DENV infection and anticipated to be discontinued [15–17]. Currently, there is no specific antiviral therapy for dengue; clinical management is supportive. Timely and accurate diagnostics are essential for patient care, outbreak control, and vaccine implementation, particularly in endemic regions with co-circulating flaviviruses. Because early symptoms of dengue are non-specific, laboratory confirmation is critical to distinguish it from other febrile illnesses and guide appropriate interventions. Diagnostic strategies should consider the stage of infection, the timing of sample collection, kinetics of viremia and antibody responses, purpose of testing, regional flavivirus circulation, prior flavivirus exposure or vaccination, travel history, and available laboratory infrastructure [18–20].

During the early febrile phase (typically within the first 5–7 days after symptom onset), DENV infection can be confirmed by virus isolation, or detection of viral RNA or antigens [19,20]. Virus isolation allows serotype identification but is rarely used due to complexity and low sensitivity [21]. Reverse transcription polymerase chain reaction (RT-PCR) offers high sensitivity and specificity and enables serotyping but is limited by the short duration of viremia and possible resource constraints. Antigen detection focuses on the non-structural protein 1 (NS1), a highly immunogenic glycoprotein secreted from infected cells during viral replication. NS1 is more stable than viral RNA, extending the diagnostic window of antigen assays beyond that of molecular methods [22–25]. However, NS1 detection in secondary infections may be compromised by the early rise in anti-NS1 IgG titers due to the anamnestic immune response. The high IgG levels lead to the formation of immune complexes, which ‘mask’ the NS1 antigen, reducing its detectability and lowering assay sensitivity. This effect is particularly pronounced in secondary infections, where immune complex formation can substantially decrease the reliability of NS1-based diagnostic during the acute phase of infection [26–28].

IgM antibodies are initially detectable between 3–5 days after symptom onset and remain for several months following primary DENV infection. IgG antibodies usually appear by day 7–10 and can persist for years. In secondary flavivirus infections, the IgM response is typically altered, while IgG titers rise rapidly and reach high levels due to prior immunological priming [13,19,21]. As per WHO guidelines, a single positive IgM result indicates a probable dengue case, while IgM or IgG seroconversion, or a 4-fold rise in IgG titers in paired (acute and convalescent) samples confirms recent DENV infection [21,29,30]. In endemic areas, where molecular diagnostics are often unavailable, single-sample serology is commonly used despite its limitations, as paired samples are difficult to obtain due to short hospital stays and high outpatient loss to follow-up.

Interpretation of serological results is complicated by extensive cross-reactivity among flaviviruses, especially in populations with frequent prior infections or vaccination history [31,32]. Many assays available for the measurement of IgM or IgG antibodies are based on native whole-virus antigens or recombinant proteins, such as envelope glycoprotein E (gE). These assays detect broadly reactive antibodies, which limits their ability to differentiate between flavivirus infections [33–36]. To improve specificity, assays employing recombinant antigens have been developed, with NS1 serving as a key target for antibody detection. Containing more species-specific epitopes than structural proteins, NS1 allows better distinction among flavivirus infections, although some cross-reactivity remains [37–42]. Importantly, NS1 is absent in several inactivated flavivirus vaccines (e.g., TBEV, JEV), enabling possible distinction between vaccination responses and natural infections [37,43].

Given the limitations of individual diagnostic markers, a combined approach that integrates the determination of viral RNA, viral antigens, and host antibodies is recommended [19,20]. As molecular testing is not universally available in resource-limited settings, robust serological assays remain essential. Addressing this need, we assessed the diagnostic accuracy of a set of ELISA kits for DENV serodiagnosis provided by EUROIMMUN Medizinische Labordiagnostika AG (Lübeck, Germany; hereafter “Euroimmun”). We evaluated these assays individually and in combination, focusing on two key aspects: [i] the added diagnostic value of combining DENV NS1 antigen and anti-DENV IgM detection for confirming acute infections, and [ii] the evaluation of the Anti-DENV NS1 ELISA 2.0 (IgG), a novel assay with a dual cut-off designed to optimize serological interpretation in flavivirus-endemic and non-endemic regions.

## Methods

### Ethics statement

The study was conducted in accordance with the Declaration of Helsinki in its current version. The DENV samples originated from a previously published study [44], which was approved by the Institutional Review Board of Vietnam Military Medical University (VMMU), Hanoi, Vietnam (Nr. 103MCH/RES/DENV-GER_V-D1-2016) and the 108 Hospital (Nr. 108MCH/RES/DENV_D1-08-05-2018-SDL); written informed consent was obtained from all recruited dengue patients [44]. The ZIKV samples were collected under IRB Protocol No. 6874, which underwent review by the CDC IRB and was deemed exempt. For child participants in this ZIKV cohort, written informed consent was obtained from a parent or legal guardian prior to enrollment. In addition, assent was obtained from children when appropriate according to age and local regulations; verbal assent was obtained from younger children, and written assent from older minors. The CHIKV samples, provided by Cerba Xpert (Frépillon, France), were fully anonymized leftovers from clinical laboratories; therefore consent was not necessary. According to Cerba Xpert’s ethical and regulatory framework, patients may opt out of having their data and samples used for purposes other than their personal care; for child participants, a parent or legal guardian may exercise the opt‑out option. The provision of additional biospecimens from commercial suppliers was also in compliance with the relevant ethical and legal standards. All samples in this study were pseudonymized or anonymized at the participating sites following local institutional guidelines and transferred under coded or anonymized identifiers only. The pseudonymization key remained exclusively at the originating institutions and was never disclosed, ensuring that it is not possible to re-identify the samples and that all material is therefore transferred in an effectively anonymized manner.

### Patients and samples

Demographic and clinical characteristics of the study cohorts are presented in Table 1. All samples were stored frozen at −20°C until serological testing.

**Table 1:** Information on study cohorts and sample characteristics.

Parameter		Dengue virus (DENV)	Healthy blood donors (HBD)	West Nile virus (WNV)	Zika virus (ZIKV)	Chikungunya virus (CHIKV)
**Patient demographics**						
	Number, *n*	22	500	40	40	50
	Residence	Vietnam	Germany	USA	Puerto Rico	FOTs ^a^, France
	Mean age ± SD (range), years	33.6 ± 10.4 (19-59)	35.0 ± 12.4 (18-69)	―	12.1 ± 2.2 (9-16)	37.5 ± 6.4 (14-50)
	Female/male, *n*	11/11	122/378	16/24	15/25	36/14
**Clinical signs, *n* (%)** ^**b**^						
	Fever	19 (100%)	―	―	―	―
	Headache	19 (100%)	―	―	―	―
	Body ache	19 (100%)	―	―	―	―
	Bleeding	1 (5.3%)	―	―	―	―
	Vomiting	4 (21.1%)	―	―	―	―
	Fatigue	6 (31.6%)	―	―	―	―
	Rash	15 (78.9%)	―	―	―	―
	Petechiae	2 (10.5%)	―	―	―	―
**Clinical severity, *n* (%)**						
	Mild dengue fever	16 (72.7%)	―	―	―	―
	Moderate dengue fever	6 (27.3%)	―	―	―	―
**Pre-characterization (molecular/serological), *n* (%)**						
	DENV RT-PCR pos	22 (100%)	―	―	―	―
	DENV FRNT pos	―	―	―	0 (0%)	―
	WNV RT-PCR pos	―	―	40 (100%)	―	―
	Anti-WNV IgM ELISA pos	―	―	40 (100%)	―	―
	Anti-WNV IgG ELISA pos	―	―	30 (75.0%)	―	―
	Anti-WNV NS1 IgG ELISA pos	―	―	―	1 (2.5%)	―
	ZIKV RT-PCR pos	0 (0%)	―	―	―	―
	ZIKV FRNT pos	―	―	―	40 (100%)	―
	Anti-ZIKV IgM ELISA pos	0 (0%)	―	―	0 (0%)	―
	Anti-ZIKV IgG ELISA pos	1 (4.5%)	―	―	40 (100%)	―
	ZIKV EDIII IgG ELISA pos	―	―	―	20 (100%) ^c^	―
	CHIKV RT-PCR pos	0 (0%)	―	―	―	―
	CHIKV PRNT pos	―	―	―	―	50 (100%)
	Anti-CHIKV IgM ELISA pos	―	―	―	―	47 (94.0%)
	Anti-CHIKV IgG ELISA pos	―	―	―	―	50 (100%)
	YFV RT-PCR pos	0 (0%)	―	―	―	―
	*Plasmodium* PCR pos	0 (0%)	―	―	―	―
**DENV serotype, *n* (%)**						
	DENV1	15 (68.2%)	―	―	―	―
	DENV2	6 (27.3%)	―	―	―	―
	DENV1 and DENV2	1 (4.5%)	―	―	―	―
**Samples**						
	Type	Plasma	Plasma	Plasma	Serum	Serum
	Total number, *n*	63^d^	500	40	40	50
**t1 samples (acute phase)**						
	Number, *n*	21	―	―	―	―
	Mean dpo ± SD	3.9 ± 1.5	―	―	―	―
	Median dpo (range)	4 (1-6)	―	―	―	―
**t2 samples (early convalescent phase)**						
	Number, *n*	20	―	―	―	―
	Mean dpo ± SD	6.9 ± 1.6	―	―	―	―
	Median dpo (range)	7 (4-9)	―	―	―	―
**t3 samples (late convalescent phase)**						
	Number, *n*	22	―	―	―	―
	Mean dpo ± SD	16.4 ± 1.9	―	―	―	―
	Median dpo (range)	17 (13-19)	―	―	―	―

dpo, days post onset; ELISA, enzyme-linked immunosorbent assay; FOTs, French Overseas Territories; FRNT, focus reduction neutralization test; IgG, immunoglobulin G; IgM, immunoglobulin M; PRNT, plaque reduction neutralization test; RT-PCR, real-time reverse transcription polymerase chain reaction; SD, standard deviation; YFV, yellow fever virus; ―, data not available or not determined.

^a^Martinique, Guadeloupe, French Guiana, and Réunion.

^b^Clinical signs were available for only 19 of 22 DENV-infected patients; only a subset of signs is shown.

^c^Only 20 of 40 ZIKV samples were tested with the ZIKV EDIII IgG ELISA; all tested samples were positive.

^d^Three samples were collected from each of the 22 patients; 63 of 66 samples were available in sufficient quantity for serological testing in this study.

#### Dengue panel.

Plasma samples were collected from 22 hospitalized Vietnamese patients with mild to moderate dengue fever during a seasonal DENV outbreak in Hanoi, Vietnam, in 2017 [44]. Clinical and laboratory diagnosis followed the revised dengue case definitions issued by the World Health Organization (WHO) in 2009 [30]. Samples were provided by SeraDiaLogistics (Munich, Germany).

Three sequential plasma samples were obtained from each patient, yielding 66 samples, 63 of which were available in sufficient quantity for serological testing. The first sample was drawn on the day of hospital admission, within 6 days after reported onset of fever (sample group t1: acute viremic phase). The second sample was collected 3 days after hospitalization (t2: early convalescent phase), and the third sample another 7–11 days later (t3: late convalescent phase; Table 1).

All t1 samples were tested by RT-PCR at SeraDiaLogistics using the LightMix Modular Dengue Virus and LightMix Modular Dengue Typing kits (TIB Molbiol, Berlin, Germany; distributed by Roche Diagnostics, Basel, Switzerland), confirming DENV infection in all patients (22/22, 100%). The infecting serotype was DENV-1 in 15/22 (68.2%), DENV-2 in 6/22 (27.3%), and a DENV-1/DENV-2 co-infection in 1/22 (4.5%) cases. Patients were not classified according to primary or secondary DENV infection status, as the ELISAs applied in this study are not intended to be used for this purpose and because relevant patient history was not available. Co-infections with ZIKV, CHIKV, YFV, or *Plasmodium* spp. were excluded by negative PCR results (Altona Diagnostics, Hamburg, Germany) at t1. Prior ZIKV infection was ruled out in 21/22 cases, based on negative Anti-ZIKV ELISA (IgG, IgM; Euroimmun) reactivity at t1.

#### Healthy control panel.

The study included 500 plasma samples from healthy blood donors (HBD) aged 18–69 years, residing in Northern Germany, where DENV and most other flaviviruses are not endemic [8]. Samples were collected at the University Medical Center Schleswig-Holstein (Lübeck, Germany) and were neither subjected to molecular testing for arboviral infections nor selected based on any predefined criteria.

#### WNV panel.

Forty plasma samples were obtained from U.S. patients with acute WNV infection (Plasma Services Group, Moorestown, NJ, USA). Acute infection was confirmed by PCR (UltraQual West Nile Virus RT-PCR; National Genetics Institute, Los Angeles, CA, USA) and by anti-WNV IgM positivity; anti-WNV IgG reactivity was additionally determined (West Nile Virus IgM Capture Dx Select ELISA, West Nile Virus IgG Dx Select ELISA; Focus Diagnostics, Cypress, CA, USA).

#### ZIKV panel.

Forty serum samples were collected from healthy Puerto Rican children aged 9–16 years during serosurveys after the ZIKV epidemic in 2015–2017 [45]. Samples were provided by the Centers for Disease Control and Prevention (CDC) Dengue Branch (San Juan, Puerto Rico, USA). All ZIKV cases were classified using the ZIKV EDIII IgG ELISA and FRNT_50_ as previously described [42]. All samples showed high ZIKV-neutralizing antibody titers (FRNT_50_ > 80) but no neutralizing activity against the four DENV serotypes. Sampling was performed well after ZIKV infection, as indicated by anti-ZIKV IgM negativity and IgG positivity.

#### CHIKV panel.

Fifty serum samples were obtained from CHIKV-infected patients, including 40 residents of the French Overseas Territories (FOTs) and 10 from mainland France. Samples were collected during the 2014–2015 CHIKV outbreak [46] and provided by Cerba Xpert (Frépillon, France). In-house ELISAs (Cerba Xpert) detected anti-CHIKV IgM and IgG in 47/50 and 50/50 samples, respectively, confirmed by a CHIKV plaque reduction neutralization test (PRNT) at the University of Padova, Italy.

### ELISAs

Samples were tested using ELISA kits from Euroimmun, following the manufacturer’s instructions: [i] Dengue Virus NS1 ELISA, [ii] Anti-Dengue Virus Type 1–4 ELISA (IgM), [iii] Anti-Dengue Virus Type 1–4 ELISA (IgG), and [iv] Anti-Dengue Virus NS1 ELISA 2.0 (IgG). Assay characteristics and recommendations for result interpretation are given in S1 Table. Each test run included kit-provided calibrators and controls. ELISA testing was performed by laboratory personnel blinded to PCR results and infection status. Optical density (OD) values were automatically recorded and converted into final results.

IgM reactivity was assessed semiquantitatively by calculating the ratio of the sample OD to the calibrator OD. NS1 and IgG levels were determined quantitatively in relative units per milliliter (RU/mL) using calibration curves. Samples with results outside the measurement range were not routinely retested. Instead, results were set to the respective lower or upper limit and displayed accordingly in all analyses and plots: DENV NS1 ELISA results >100 RU/mL were set to 100 RU/mL, Anti-DENV Type 1–4 ELISA (IgG) <2 RU/mL to 2 RU/mL and >200 RU/mL to 200 RU/mL, and Anti-DENV NS1 ELISA 2.0 (IgG) <1 RU/mL to 1 RU/mL and >80 RU/mL to 80 RU/mL.

For the Anti-DENV NS1 ELISA 2.0 (IgG), an alternative cut-off of 20 RU/mL (instead of the standard cut-off 10 RU/mL) may be applied for testing in flavivirus-endemic regions.

### Statistics

Statistical analyses and graph generation were performed using GraphPad Prism version 10.3.1 (GraphPad Software; Inc., San Diego, CA, USA) and SigmaPlot version 13.0 (Systat Software Inc., San Jose, CA, USA). Sensitivity was calculated as the proportion of positive results among samples from PCR-confirmed dengue patients. Specificity was calculated as the proportion of negative results among samples included in the specificity panel, which comprised specimens from German HBD and individuals with confirmed non-dengue infections. Borderline results were considered negative for diagnostic accuracy analysis to avoid overestimation of sensitivity.

Confidence intervals (95% CI) were calculated using the Clopper-Pearson method. Overall differences across time points were assessed with the Friedman test. If significant, post-hoc pairwise comparisons were performed using paired *t*-tests or Wilcoxon signed-rank tests, depending on the normality of paired differences (Shapiro-Wilk test). Resulting *p*-values were adjusted for multiple comparisons using the Bonferroni method. *p*-values <0.05 were considered statistically significant.

## Results

### Sensitivity of NS1, IgM, and IgG ELISAs in acute and convalescent stages

Sensitivities of the Euroimmun ELISAs for DENV NS1, anti-DENV IgM, and anti-DENV IgG were determined using DENV RT-PCR as the reference standard, based on qualitative results obtained at three defined sampling time points (t1-t3, Table 2).

**Table 2:** Qualitative assay results and sensitivities at sampling time points t1, t2, and t3 in 22 patients with PCR-confirmed DENV infection.

Sampling time: range	*n*	Metric	DENV RT-PCR (reference)	DENV NS1 ELISA	Anti-DENV Type 1–4 ELISA (IgM)	Anti-DENV Type 1–4 ELISA (IgG)	Anti-DENV NS1 ELISA 2.0 (IgG)^c^	
							Standard cut-off	Alternative cut-off
**t1:** **1-6 dpo**	21^a^	Pos/bdl/neg	21/0/0	19/0/2	7/2/12	14/1/6	7/0/14	4/1/16
		Sensitivity (%)^b^	NA	90.5%	33.3%	66.7%	33.3%	19.1%
		[95% CI]	NA	[69.6, 98.8]	[14.6, 57.0]	[43.0, 85.4]	[14.6, 57.0]	[5.5, 41.9]
**t2:** **4-9 dpo**	20^a^	Pos/bdl/neg	ND	14/0/6	17/2/1	20/0/0	13/1/6	10/1/9
		Sensitivity (%)^b^	ND	70.0%	85.0%	100%	65.0%	50.0%
		[95% CI]	ND	[45.7, 88.1]	[62.1, 96.8]	[83.2, 100]	[40.8, 84.6]	[27.2, 72.8]
**t3:** **13-19 dpo**	22	Pos/bdl/neg	ND	0/0/22	17/1/4	22/0/0	22/0/0	22/0/0
		Sensitivity (%)^b^	ND	0%	77.3%	100%	100%	100%
		[95% CI]	ND	[0, 15.4]	[54.6, 92.2]	[84.6, 100]	[84.6, 100]	[84.6, 100]

Bdl, borderline; CI, confidence interval; DENV, dengue virus; dpo, days post onset; ELISA, enzyme-linked immunosorbent assay; IgG, immunoglobulin G; IgM, immunoglobulin M; neg, negative; NA, not applicable; ND, not determined at this time point; NS1, non-structural protein 1; pos, positive; RT-PCR, real-time reverse transcription polymerase chain reaction.

^a^ Three samples (t1: n = 1; t2: n = 2) were excluded because they were not available in sufficient quantity for serological testing in this study.

^b^ Borderline results were considered negative for sensitivity calculations.

^c^An alternative cut-off (20 RU/mL) may be applied for samples from flavivirus-endemic areas, instead of the standard cut-off (10 RU/mL).

At t1 (1–6 dpo), the DENV NS1 ELISA demonstrated the highest sensitivity at 90.5% (19/21), consistent with the presence of circulating NS1 antigen during early infection. Sensitivity decreased to 70.0% (14/20) at t2 (4–9 dpo) and dropped to 0% (0/22) at t3 (13–19 dpo), reflecting the transient nature of NS1 antigenemia [47].

The Anti-DENV Type 1–4 ELISA (IgM) showed a sensitivity of 33.3% (7/21) at t1, increasing to 85.0% 17/20) at t2, before slightly declining to 77.3% (17/22) at t3. This pattern reflects typical kinetics of IgM seroconversion following acute DENV infection.

Both IgG ELISAs exhibited an upwards trend in positivity rates across all three time points. The Anti-DENV Type 1–4 ELISA (IgG), which uses a native antigen (highly purified virus particles) and recombinant envelope glycoprotein E (gE) as substrate, achieved a sensitivity of 66.7% (14/21) at t1 and reached 100% (20/20) by t2, maintaining this level at t3. The rapid attainment of full sensitivity suggests robust detection performance, likely attributable to the assay’s broad antigenic coverage.

In contrast, the Anti-DENV NS1 ELISA 2.0 (IgG) showed a slower increase in positivity rates. Using the standard (10 RU/mL) versus alternative (20 RU/mL) cut-off, sensitivity amounted to 33.3% (7/21) versus 19.1% (4/21) at t1, and 65.0% (13/20) versus 50.0% (10/20) at t2, respectively, reaching 100% (22/22) only at t3.

### Kinetics of NS1, IgM and IgG

**Fig 1:**
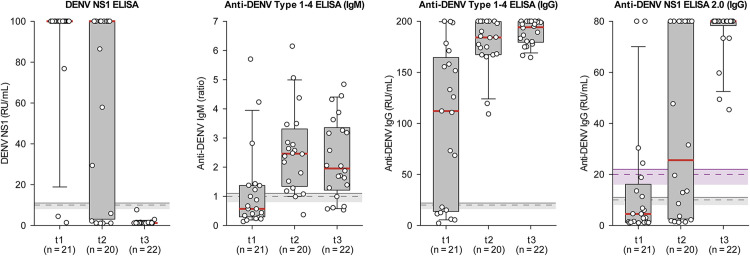
Distribution of DENV NS1, anti-DENV IgM and anti-DENV IgG levels measured with the indicated ELISAs. Samples were collected from 22 dengue patients at time points t1 (1-6 days post onset, dpo), t2 (4-9 dpo) and t3 (13-19 dpo). Boxes indicate interquartile ranges (outer bounds) and medians (bold red lines). Whiskers present the 90th and 10th percentiles. In each graph, the dashed line shows the assay-specific cut-off, the adjacent shaded area indicates the borderline range, and the solid horizontal line marking the upper limit of the borderline range represents the positivity threshold. For the Anti-DENV NS1 ELISA 2.0 (IgG), an alternative cut-off value (20 RU/mL, purple) may be applied for samples from flavivirus-endemic areas, instead of the standard cut-off (10 RU/mL, gray).

Temporal dynamics of DENV markers (NS1, IgM, and IgG) were assessed across t1, t2, and t3 based on quantitative ELISA results, revealing distinct distributions over time (Fig 1). NS1 and IgG showed an inverse kinetic pattern from acute infection to convalescence. NS1 levels were highest at t1 and declined progressively at t2 and t3. This trend was reflected by a decreasing median and a narrowing interquartile range (IQR) between t2 and t3, indicating a uniform clearance of circulating antigen during the transition from acute to convalescent stages across the cohort.

In contrast, IgG levels increased over time in both IgG ELISAs, with rising median values and narrowing IQRs, particularly in the Anti-DENV Type 1–4 ELISA (IgG). The Anti-DENV NS1 ELISA 2.0 (IgG) exhibited delayed detection dynamics with lower median values at t1 and t2, and a broader IQR at t2, reflecting heterogeneous IgG responses among patients. At t3, however, IgG reactivity measured with this assay was uniformly high, as indicated by a narrow IQR and a high median value at the upper detection limit (80 RU/mL). This pattern suggests that by t3, most patients had mounted a strong IgG response detectable even with the assay’s selective antigen design. The delay is likely due to the later onset of anti-NS1 IgG production compared to IgG responses against whole-virus antigens.

IgM levels peaked at t2, with a broader IQR suggesting inter-individual variability in seroconversion timing. At t3, IgM responses showed a slight decline or plateau.

**Fig 2:**
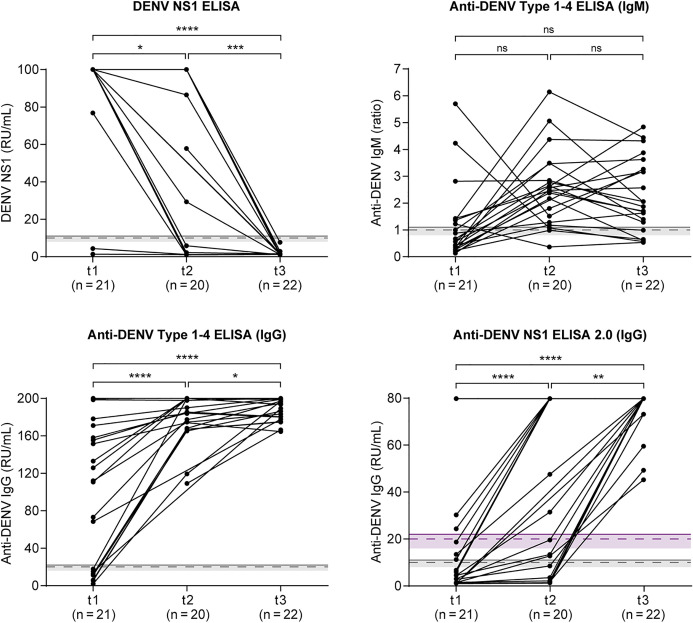
Kinetics of DENV NS1, anti-DENV IgM and anti-DENV IgG measured in 22 dengue patients across three time points: t1 (1-6 days post onset, dpo), t2 (4-9 dpo) and t3 (13-19 dpo). For three patients, samples were available in sufficient quantity only from two time points. *p*-values were calculated for pairwise comparisons using the paired *t*-test if differences between paired values were normally distributed (Shapiro-Wilk test); otherwise, the Wilcoxon signed-rank test was performed. A Bonferroni correction was applied to adjust for multiple comparisons (*n* = 3). Statistical significance was defined as *p* < 0.05 after Bonferroni adjustment. Asterisks denote significance levels: ns, not significant; * *p* < 0.05; ** *p* < 0.01; *** *p* < 0.001; **** *p* < 0.0001. For details on graphical elements (dashed lines, shaded areas, and thresholds), see Fig 1.

These descriptive trends were further supported by statistical analysis (Fig 2). NS1 levels declined significantly over time, with pairwise comparisons showing increasing statistical strength: t1 vs. t2 (p = 0.023), t2 vs. t3 (p = 1.5 × 10 ⁻ ⁴), and t1 vs. t3 (p = 2.9 × 10 ⁻ ⁶). IgG reactivity increased significantly over time in both ELISAs. For the Anti-DENV Type 1–4 ELISA (IgG), significant differences were observed between t1 and t2 (p = 6.9 × 10 ⁻ ⁵), t2 and t3 (p = 0.047), and t1 and t3 (p = 5.7 × 10 ⁻ ⁶). The Anti-DENV NS1 ELISA 2.0 (IgG) showed similar trends: t1 vs. t2 (p = 4.6 × 10 ⁻ ⁵), t2 vs. t3 (p = 0.0015), and t1 vs. t3 (p = 1.1 × 10 ⁻ ⁵). Notably, the inverse kinetic relationship between NS1 and IgG was more evident in measurements with the anti-NS1 IgG ELISA. IgM changes were less pronounced and did not reach statistical significance (t1 vs. t2: p = 0.072; t2 vs. t3: p = 0.236; t1 vs. t3: p = 0.076).

Chronological sorting of all 63 samples by dpo revealed that NS1 antigen remained detectable up to day 9. The Anti-DENV Type 1–4 ELISA (IgG) reached a positivity rate of 100% from day 7 onwards, with all subsequent samples testing positive. In contrast, the Anti-DENV NS1 ELISA 2.0 (IgG) did not achieve full sensitivity until day 13 after symptom onset (S2 Table). These results illustrate the relatively prolonged detectability of NS1 antigen, extending beyond the typical viremic period. In addition, they reflect distinct temporal windows of effective antibody detection for the two IgG ELISAs.

Seromarker kinetics across grouped dpo intervals and individual longitudinal profiles of all 22 patients are presented in S1 and S2 Figs, respectively. These data further illustrate the temporal patterns of marker levels and the variability in individual serological responses. Patient #7 showed atypical longitudinal courses (S2 Fig) with high IgG levels at t1 (3 dpo) and early IgM decline in the absence of NS1 antigen, possibly indicating a secondary infection or a longer interval between symptom onset and initial sampling than recorded.

### ELISA performance on single acute-phase samples: added value of combined NS1 and IgM testing

Acute-phase (t1) samples were available from 21 patients with PCR-confirmed DENV infection. The Euroimmun DENV NS1 ELISA yielded positive results in 19/21 (90.5%) samples. IgM reactivity, assessed using the Euroimmun Anti-DENV Type 1–4 ELISA (IgM), was negative or borderline in 14/21 (66.7%) samples, all of which were NS1 positive. IgM positivity was detected in 7/21 (33.3%) samples. Notably, two of these IgM-positive samples (collected at 3 and 6 dpo) were the only NS1-negative cases. Both tested positive for anti-DENV IgG using the Euroimmun Anti-DENV Type 1–4 ELISA (IgG) as well as the Anti-DENV NS1 ELISA 2.0 (IgG), regardless of whether standard or alternative cut-offs were applied. By combining NS1 and IgM ELISAs, the sensitivity gaps observed with the individual assays were fully compensated, resulting in a combined detection rate of 100% (21/21; Table 3). Although all RT-PCR-positive cases were detected in this cohort, the small sample size provides only limited statistical robustness; therefore, these results should be interpreted with caution and cannot be directly generalized to broader populations.

**Table 3:** Qualitative ELISA results for detection of DENV NS1, anti-DENV IgM and anti-DENV IgG in acute-phase samples (t1) from 21 patients with PCR-confirmed DENV infection, sorted by days post onset (dpo).

Sampling time (dpo)	Patient No.^a^	DENV RT-PCR (reference)	DENV NS1 ELISA	Anti-DENV Type 1–4 ELISA (IgM)	Combined positivity NS1/IgM^b,c^	Anti-DENV Type 1–4 ELISA (IgG)	Anti-DENV NS1 ELISA 2.0 (IgG)^d^	
							Standard cut-off	Alternative cut-off
**1**	9	pos	pos	pos	+	neg	neg	neg
**2**	3	pos	pos	neg	+	pos	neg	neg
**2**	14	pos	pos	neg	+	pos	neg	neg
**2**	16	pos	pos	neg	+	pos	neg	neg
**3**	1	pos	pos	pos	+	pos	pos	pos
**3**	6	pos	pos	*bdl*	+	pos	pos	neg
**3**	7	pos	neg	pos	+	pos	pos	pos
**3**	11	pos	pos	neg	+	neg	neg	neg
**3**	13	pos	pos	neg	+	pos	pos	neg
**3**	20	pos	pos	pos	+	pos	neg	neg
**4**	5	pos	pos	neg	+	pos	neg	neg
**4**	22	pos	pos	*bdl*	+	pos	pos	pos
**5**	2	pos	pos	neg	+	*bdl*	neg	neg
**5**	8	pos	pos	pos	+	pos	neg	neg
**5**	10	pos	pos	pos	+	pos	pos	*bdl*
**5**	17	pos	pos	neg	+	neg	neg	neg
**5**	18	pos	pos	neg	+	neg	neg	neg
**6**	12	pos	pos	neg	+	neg	neg	neg
**6**	15	pos	pos	neg	+	pos	neg	neg
**6**	19	pos	neg	pos	+	pos	pos	pos
**6**	21	pos	pos	neg	+	neg	neg	neg
***n* (tested samples)** ^**a**^		21	21	21	21	21	21	21
**Pos/bdl/neg**		21/0/0	19/0/2	7/2/12	21/21	14/1/6	7/0/14	4/1/16
**Sensitivity (%)** ^**c**^		NA	90.5%	33.3%	100%	66.7%	33.3%	19.1%
**[95% CI]**		NA	[69.6, 98.8]	[14.6, 57.0]	[83.9, 100]	[43.0, 85.4]	[14.6, 57.0]	[5.5, 41.9]

Bdl, borderline; CI, confidence interval; DENV, dengue virus; dpo, days post onset; ELISA, enzyme-linked immunosorbent assay; IgG, immunoglobulin G; IgM, immunoglobulin M; NA, not applicable; neg, negative; NS1, non-structural protein 1; pos, positive; RT-PCR, real-time reverse transcription polymerase chain reaction.

^a^Acute-phase samples were available from only 21/22 dengue patients. One t1 sample (patient #4) was excluded because it was not available in sufficient quantity for serological testing in this study.

^b^Combined positivity (+) was defined as positive reactivity in at least one of the two ELISAs (DENV NS1 or anti-DENV IgM).

^c^Borderline results (bdl) were considered negative for sensitivity calculations.

^d^For the Anti-DENV NS1 ELISA 2.0 (IgG), an alternative cut-off value (20 RU/mL) may be applied for samples from flavivirus-endemic areas, instead of the standard cut-off (10 RU/mL).

### Paired-sample assessment of IgM/IgG seroconversion and IgG increase

To assess ELISA performance in identifying recent DENV infections using paired samples, we analyzed: [i] anti-DENV IgM/IgG seroconversion (defined as a change from an IgM-negative acute sample to an IgM-positive early or late convalescent sample and/or from an IgG-negative acute sample to an IgG-positive early or late convalescent sample); and [ii] ≥4-fold increases in IgG levels between the acute sample and at least one of the convalescent samples. These criteria are widely recognized indicators of recent infection [30] and are commonly applied in epidemiological studies and retrospective case confirmation, especially when molecular or antigen detection methods are unavailable, inconclusive, or performed outside the optimal diagnostic window.

IgM seroconversion between t1 and t2 and/or between t1 and t3 was observed in 13/21 (61.9%) dengue patients. Among the 8 patients without detectable IgM seroconversion, 7 tested IgM-positive already in the acute phase (t1), while 1 patient remained IgM-negative or -borderline throughout the sampling period as could occur in a secondary flavivirus infection (Table 4).

**Table 4:** IgM/IgG seroconversion and IgG increase between acute-phase samples (t1) and convalescent-phase samples (t2, t3) from 21 patients with PCR-confirmed DENV infection. Gray-shaded cells indicate cases where IgM and IgG results suggest recent DENV infection.

			IgM seroconversion between t1 and t2 and/or t1 and t3 *Anti-DENV Type 1–4 ELISA (IgM)*		
**Detected**	**Not detected^b^**	**Total**
**IgG seroconversion and/or ≥4-fold increase in IgG levels between t1 and t2 and/or t1 and t3**	***Anti-DENV Type 1–4 ELISA (IgG)***	Detected	6	1	7
		Not detected ^b^	7	7	14
		Total	13	8	21^a^
	***Anti-DENV NS1 ELISA 2.0 (IgG); alternative cut-off***	Detected	13	8	21
		Not detected ^b^	0	0	0
		Total	13	8	21^a^

DENV, dengue virus; ELISA, enzyme-linked immunosorbent assay; IgG, immunoglobulin G; IgM, immunoglobulin M; NS1, non-structural protein 1.

^a^One patient (#4) was excluded because the t1 sample was not available in sufficient quantity for serological testing in this study.

^b^Borderline results were considered negative for seroconversion analysis.

IgG seroconversion and/or a ≥ 4-fold increase was detected in 7/21 (33.3%) patients using the Anti-DENV Type 1–4 ELISA (IgG), including 6 cases with concomitant IgM seroconversion. For 4 patients (#1, #7, #19 and #22), IgG concentrations in the Anti-DENV NS1 ELISA 2.0 (IgG) exceeded the assay’s upper limit of quantification (80 RU/mL), which prevented assessment of ≥4-fold increases based on the initial measurements (see S2 Fig for original non-titrated IgG values). To overcome this limitation, the samples of these patients were titrated using the Anti‑DENV NS1 ELISA 2.0 (IgG), allowing accurate quantification of IgG levels. When applying the titrated measurements, IgG seroconversion and/or ≥4‑fold increases were observed in 21/21 (100%) patients, of whom 13 also had concomitant IgM seroconversion (Table 4).

Combining IgM and IgG results from paired specimens allowed confirmation of recent DENV infection in 14/21 (66.7%) of patients including the results of the Anti-DENV Type 1–4 ELISA (IgG). This rate increased to 21/21 (100%) when IgG was assessed using the Anti-DENV NS1 ELISA 2.0 (IgG).

### Specificity of anti-DENV IgG ELISAs

The specificity of the IgG ELISAs was determined using samples from HBD from a flavivirus non-endemic country, and from patients with confirmed exposure to WNV and ZIKV. The ZIKV panel comprised pediatric samples in which the absence of DENV-specific neutralizing antibodies was confirmed by NT, the recognized gold standard for serological specificity in flavivirus diagnostics. The use of this ZIKV panel therefore provides a stringent approach to assessing assay cross-reactivity, which is a major challenge in flavivirus serology due to high antigenic similarity between ZIKV and DENV.

**Fig 3:**
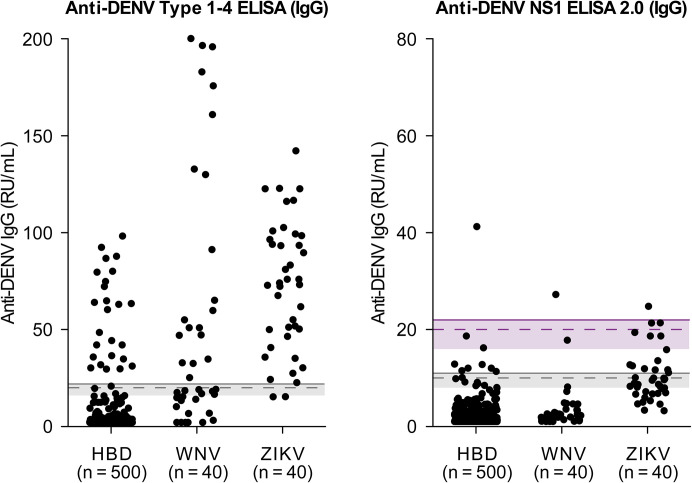
Reactivity of anti-DENV IgG ELISAs in samples from healthy blood donors (HBD) and patients infected with West Nile virus (WNV) or Zika virus (ZIKV). For details on graphical elements (dashed lines, shaded areas, and thresholds), see Fig 1.

The Anti-DENV Type 1–4 ELISA (IgG) had an overall specificity of 85.7% (497/580) (Table 5). Among HBD, 95.0% (475/500) specificity was observed, indicating detectable background reactivity even in non-endemic populations. Specificity was low to moderate in the ZIKV (5.0%, 2/40) and WNV (50.0%, 20/40) panels, with positive samples frequently showing high IgG reactivity (Fig 3, Table 5).

**Table 5:** Specificity of anti-DENV IgG ELISAs.

Panel	*n*	Metric	Anti-DENV Type 1–4 ELISA (IgG)	Anti-DENV NS1 ELISA 2.0 (IgG)^b^	
				Standard cut-off	Alternative cut-off
**Healthy blood donors (HBD)**	500	Pos/bdl/neg	25/3/472	9/10/481	1/2/497
		Specificity (%)^a^	95.0%	98.2%	99.8%
		[95% CI]	[92.7, 96.7]	[96.6, 99.2]	[98.9, 100]
**West Nile virus (WNV)**	40	Pos/bdl/neg	20/7/13	2/1/37	1/1/38
		Specificity (%)^a^	50.0%	95.0%	97.5%
		[95% CI]	[33.8, 66.2]	[83.1, 99.4]	[86.8, 99.9]
**Zika virus (ZIKV)**	40	Pos/bdl/neg	38/0/2	14/12/14	1/5/34
		Specificity (%)^a^	5.0%	65.0%	97.5%
		[95% CI]	[0.6, 16.9]	[48.3, 79.4]	[86.8, 99.9]
**Total**	580	Pos/bdl/neg	83/10/487	25/23/532	3/8/569
		Specificity (%)^a^	85.7%	95.7%	99.5%
		[95% CI]	[82.6, 88.4]	[93.7, 97.2]	[98.5, 99.9]

Bdl, borderline; CI, confidence interval; DENV, dengue virus; ELISA, enzyme-linked immunosorbent assay; IgG, immunoglobulin G; IgM, immunoglobulin M; neg, negative; NS1, non-structural protein 1; pos, positive.

^a^Borderline results were considered negative for specificity calculations.

^b^For the Anti-DENV NS1 ELISA 2.0 (IgG), an alternative cut-off value (20 RU/mL) may be applied for samples from flavivirus-endemic areas, instead of the standard cut-off (10 RU/mL).

In contrast, the Anti-DENV NS1 2.0 ELISA (IgG) demonstrated higher specificity. Using the standard cut-off, overall specificity amounted to 95.7% (555/580), with 98.2% (491/500) in HBD and 95.0% (38/40) in WNV samples. Specificity in the ZIKV panel remained moderate at 65.0% (26/40), with several patients showing low or borderline IgG levels. Applying the alternative cut-off further increased overall specificity to 99.5% (577/580), with only 3 positive samples among 580 tested, reaching 99.8% (499/500) specificity in HBD and 97.5% (39/40) in both WNV and ZIKV panels (Table 5).

Compared to the Anti-DENV Type 1–4 ELISA (IgG), the Anti-DENV NS1 2.0 ELISA (IgG) showed a tighter accumulation of IgG values below the positivity threshold, particularly with the alternative cut-off. The use of this assay effectively reduced background reactivity in non-endemic samples and minimized cross-reactivity in flavivirus-exposed individuals, thereby enhancing specificity (Fig 3).

### Anti-DENV IgG testing in regions with and without co-endemicity of DENV and CHIKV

To assess anti-DENV IgG positivity in settings with different flavivirus endemicities and to address the challenges in interpreting anti-DENV IgG reactivity in the absence of clinical records or flavivirus-specific reference testing, we analyzed samples from 50 patients with NT-confirmed CHIKV infection. Of these, 40 resided in the FOTs, where DENV, CHIKV, and ZIKV co-circulate [8], leading to a strong background of DENV exposure. The remaining 10 patients were from mainland France, characterized by sporadic autochthonous transmission of DENV and CHIKV [48].

**Fig 4:**
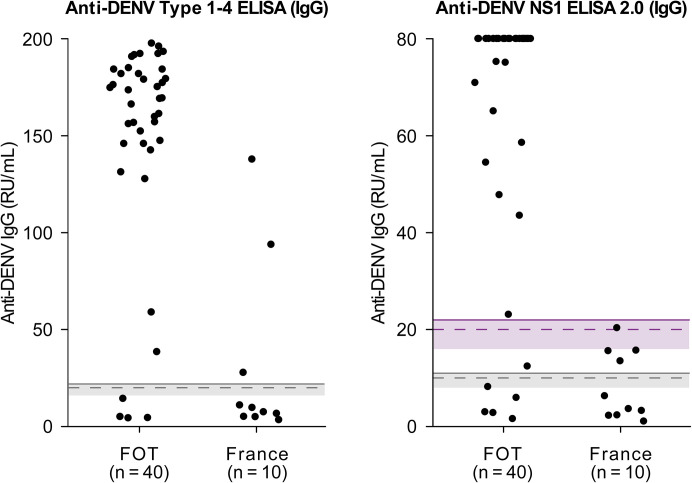
Reactivity of anti-DENV IgG ELISAs in samples from patients infected with chikungunya virus, grouped by geographic origin: flavivirus-endemic areas (French Overseas Territories, FOT) and areas with limited numbers of autochthonous cases (mainland France). For details on graphical elements (dashed lines, shaded areas, and thresholds), see Fig 1.

Among FOT patients, 90.0% (36/40) tested positive using the Anti-DENV Type 1–4 ELISA (IgG). The Anti-DENV NS1 ELISA 2.0 (IgG) yielded positivity rates of 87.5% (35/40) and 85.0% (34/40) with standard and alternative cut-offs, respectively (Table 6). Median IgG levels among positive samples were high: 174 RU/mL (range: 38–197) for the Anti-DENV Type 1–4 ELISA (IgG), and >80 RU/mL (range: 23 to >80) for the Anti-DENV NS1 ELISA 2.0 (IgG) (Fig 4). The minimal reduction in positivity when applying the higher cut-off, along with consistently high antibody levels, suggests that most IgG signals represent true anti-DENV reactivity rather than cross-reactivity.

**Table 6:** Anti-DENV IgG positivity rates among chikungunya virus-infected patients in flavivirus-endemic areas (French Overseas Territories) and areas with limited numbers of autochthonous cases (mainland France).

Area	*n*	Metric	Anti-DENV Type 1–4 ELISA (IgG)	Anti-DENV NS1 ELISA 2.0 (IgG) ^b^	
				Standard cut-off	Alternative cut-off
**FOTs**	40	Pos/bdl/neg	36/0/4	35/1/4	34/0/6
		Positivity rate (%) ^b^	90.0%	87.5%	85.0%
		[95% CI]	[76.3, 97.2]	[73.2, 95.8]	[70.2, 94.3]
**France**	10	Pos/bdl/neg	3/0/7	4/0/6	0/1/9
		Positivity rate (%) ^b^	30.0%	40.0%	0%
		[95% CI]	[6.7, 65.3]	[12.2, 73.8]	[0, 30.9]
**Total**	50	Pos/bdl/neg	39/0/11	39/1/10	34/1/15
		Positivity rate (%) ^b^	78.0%	78.0%	68.0%
		[95% CI]	[64.0, 88.5]	[64.0, 88.5]	[53.3, 80.5]

Bdl, borderline; CI, confidence interval; DENV, dengue virus; ELISA, enzyme-linked immunosorbent assay; FOTs, French Overseas Territories; IgG, immunoglobulin G; IgM, immunoglobulin M; neg, negative; NS1, non-structural protein 1; pos, positive.

^a^ Borderline results were considered negative for positivity rate calculations.

^b^ For the Anti-DENV NS1 ELISA 2.0 (IgG), an alternative cut-off value (20 RU/mL) may be applied for samples from flavivirus-endemic areas, instead of the standard cut-off (10 RU/mL).

In contrast, patients from mainland France showed lower positivity rates: 30.0% (3/10) with the Anti-DENV Type 1–4 ELISA (IgG) and 40.0% (4/10) with the Anti-DENV NS1 ELISA 2.0 (IgG) using the standard cut-off (Table 6). Median IgG reactivity was also reduced among positive samples: 93 RU/mL (range: 27–137) for the Anti-DENV Type 1–4 ELISA (IgG) and 16 RU/mL (range: 14–20) for the Anti-DENV NS1 ELISA 2.0 (IgG) (Fig 4). However, these findings should be interpreted with caution, as individual histories of prior flavivirus exposure were not available and could not be systematically assessed.

## Discussion

In this study, we analyzed the accuracy of Euroimmun ELISAs for DENV serodiagnostics during acute and convalescent phases of infection, using three sequential samples from patients with mild or moderate dengue fever. Assays targeting NS1, IgM and IgG were assessed individually or combined in algorithms for single and paired samples, with RT-PCR as reference.

Overall, the Euroimmun ELISAs reflected the expected kinetics of DENV antigen clearance and antibody development [13,47,49,50]. NS1 antigen was most prominent during the early acute phase; IgM overlapped with and extended beyond the diagnostic window of NS1, peaking during early convalescence; IgG levels rose steadily, providing robust late-stage sensitivity.

### Characteristics of ELISAs across infection stages and the impact of a dual cut-off strategy in NS1-based IgG detection

The Euroimmun DENV NS1 ELISA showed the highest positivity rate (90.5%) in acute-phase samples (1–6 dpo), consistent with previous reports about this assay (98.7-100%) [51,52] and meta-analyses (0–4 dpo: 90%, 1–7 dpo: 86%) [53]. IgM positivity measured with the Euroimmun Anti-DENV Type 1–4 ELISA (IgM) increased from 33.3% (1–6 dpo) to 85.0% (4–9 dpo), aligning with reported sensitivity ranges for this ELISA (38.1% to 98.8-100%) [51,54] and meta-analytic data (0–4 dpo: 17%, 1–7 dpo: 71%, 5–14 dpo: 82%) [53].

Specificity of the NS1 and IgM ELISAs was not addressed in this study but can be inferred from previous evaluations. Specificity values of 94.3-100% have been reported for the NS1 ELISA [51,52,55] with meta-analytic estimates of 90–93% [53]. For the IgM ELISA, specificity values amount to 91.4-100% [51,54,55] and meta-analytic estimates range from 82% to 91% [53].

IgG sensitivity reached 100% in early (4–9 dpo) and late convalescence (13–19 dpo) using the Euroimmun Anti-DENV Type 1–4 ELISA (IgG), matching previously reported detection rates for this assay (94.3%-100%) and other commercial ELISAs [51,56]. This assay demonstrated rapid seroconversion, reaching 100% positivity from day 7 onwards. Its native antigen/gE-based substrate enables broad epitope recognition, facilitating early detection of low-level IgG responses. This makes it a valuable tool for assessing exposure in suspected cases where direct pathogen detection is not feasible and paired samples can be obtained. Due to its high sensitivity and early IgG detectability, it may also support the diagnosis of secondary infections, especially in non-endemic regions. However, the broad antigenic coverage and the presence of conserved epitopes limits specificity in differential diagnostics, as it increases the likelihood of cross-reactivity with antibodies from prior flavivirus exposure or vaccination. The ELISA was 95.0% specific among German HBD, but showed substantial cross-reactivity in WNV-infected (50.0%), ZIKV-infected (95.0%) (this study), and TBEV-vaccinated individuals (32.0%) [57].

The Anti-DENV NS1 ELISA 2.0 (IgG) displayed delayed detection dynamics, reaching 100% IgG positivity only from day 13 onwards, reflecting the later onset of anti-NS1 IgG production compared to IgG responses against other antigens. Hence, this ELISA is not suitable for acute-phase diagnosis in individual suspected cases. However, its narrower antigenic focus enhances specificity, and the use of an alternative cut-off (20 RU/mL) additionally confines detection to robust IgG responses. This is highly relevant in flavivirus-endemic regions, helping to avoid false-positive results caused by low-level antibodies from past exposures with non-DENV flaviviruses. Overall specificity increased from 95.7% to 99.5% when applying the alternative cut-off, with gains in German HBD (1.6%), WNV-infected (2.5%), and ZIKV-infected patients (32.5%). In the HBD cohort, prior exposure to flaviviruses circulating to a small extent in Germany (e.g., WNV) or travel-associated arboviral infections cannot be ruled out and may have contributed to some positive results. By specifically confirming DENV infections in complex serological backgrounds, the NS1-based IgG ELISA improves differential diagnostics, facilitates serological surveillance, and may support epidemiological studies in regions with co-circulating flaviviruses. While the improved specificity is well demonstrated in this study, broader validation in diverse endemic settings is required.

### Potential use in pre-vaccination screening

Determining dengue serostatus is required before administering the Dengvaxia vaccine to prevent antibody-dependent enhancement and severe dengue in seronegative individuals after vaccination. CDC guidelines specify that IgG tests for pre-vaccination screening must achieve ≥75% sensitivity and ≥98% specificity to reliably confirm prior DENV infection. Previously, only a two-test algorithm fulfilled these criteria [42,58]. In the present study, the Anti-DENV NS1 ELISA 2.0 (IgG) achieved 100% sensitivity and 99.5% specificity with the alternative cut-off, meeting CDC requirements as a standalone assay. However, as this study does not directly assess screening performance or predictive values in asymptomatic populations, conclusions regarding its use in pre-vaccination screening remain preliminary.

The Anti-DENV NS1 ELISA 2.0 (IgG) is not validated for this purpose, but given its high specificity in convalescent samples, it could be further investigated in studies aimed at determining the serostatus prior to dengue vaccination [59,60].

### Combined NS1 and IgM serology in acute dengue: cumulative diagnostic effect

The distinct temporal profiles of NS1, IgM and IgG responses highlight the diagnostic value of combining ELISAs for accurate diagnostics throughout all stages of DENV infection. In our study, analysis of single acute-phase samples demonstrated that combining NS1 and IgM testing achieved 100% sensitivity, matching the results of RT-PCR. This supports the use of combined ELISA testing as a practical diagnostic approach, although similar detection rates in this limited cohort do not imply clinical equivalence to RT-PCR. It is also important to note, that IgM positivity in a single acute-phase sample (as observed in two cases) should be interpreted with caution. While suggestive of recent DENV exposure, it is not confirmatory and requires additional testing, such as RT-PCR or IgG serology in sample pairs [19]. Previous studies have also substantiated the diagnostic advantage of combined NS1 and IgM testing during early DENV infection (≤10 dpo) [61–65]. Incorporating IgM into the diagnostic algorithm not only extends the diagnostic window but is especially important in secondary infections, where rapid antibody responses may mask or clear circulating NS1 antigen, often already within the first few days post symptom onset due to prompt IgG production [26,27]. The formation of immune complexes was also reflected in our measurements, showing an inverse kinetic pattern of decreasing NS1 antigen and increasing anti-NS1 IgG. NS1 was undetectable beyond t2, consistent with its reported clearance by day 14 post onset in primary dengue infection [47].

### Retrospective confirmation of DENV infection using paired-sample serology

Our evaluation of seroconversion and IgG dynamics in paired specimens demonstrates the ELISAs’ ability to confirm recent DENV infections, particularly when molecular or antigen detection methods are unavailable or inconclusive. IgM seroconversion was observed in 61.9% of patients. However, its diagnostic value was limited in cases with pre-existing IgM positivity in acute-phase samples (7/21), potentially due to advanced sampling days or secondary infections.

In this paired-sample approach, the Anti-DENV NS1 ELISA 2.0 (IgG) outperformed the Anti-DENV Type 1–4 ELISA (IgG), detecting IgG seroconversion and/or a ≥ 4-fold increase in 100% vs. 33.3% of patients, respectively. The lower seroconversion rate determined with the Type 1–4 ELISA can largely be attributed to the high proportion of samples already IgG-positive at the first time point (t1), which precluded documentation of seroconversion in these individuals. The Type 1–4 ELISA, based on broadly reactive native antigen/gE, may yield high IgG levels in the acute phase due to cross-reactivity, limiting its suitability for paired-sample analyses. In contrast, the Anti-DENV NS1 ELISA 2.0 (IgG) more accurately reflects IgG dynamics by specifically targeting anti-NS1 antibodies, which typically rise during the convalescent phase. Notably, the NS1-based ELISA captured more cases without IgM seroconversion than the Type 1–4 ELISA, indicating superior performance in identifying recent infections during later stages.

Overall, combining IgM and IgG results from seroconversion and 4-fold-increase analyses in paired samples confirmed recent DENV infection in 100% of cases when using the Anti-DENV NS1 ELISA 2.0 (IgG). Two patients (#7 and #19) may represent secondary infections, given their negative NS1 results and high anti-NS1 IgG levels at t1 [50]. Early detection of IgG (<6 dpo) is typically associated with prior flavivirus exposure. Moreover, NS1 antigen sensitivity is reduced in secondary infections due to rapid immune complex formation and subsequent early clearance of circulating NS1, as discussed above. These analytically true-negative NS1 results may therefore be clinically misleading if NS1 is used as the sole diagnostic marker [26]. These findings support the use of the Euroimmun ELISAs for serological follow-up, highlighting the Anti-DENV NS1 ELISA 2.0 (IgG) as a robust tool for retrospective diagnostics and surveillance. Although ELISAs are not routinely used at the point of care in resource-limited settings, they are applied in centralized laboratories to complement rapid tests, particularly when higher diagnostic accuracy is required for surveillance, confirmation, or research purposes.

### Interpretation of anti-DENV IgG serology in co-endemic and non-endemic settings

Our findings highlight the necessity of interpreting anti-DENV IgG serology within the regional epidemiological context, given the differing flavivirus endemicity across settings. In the FOTs, characterized by co-circulation of DENV, CHIKV, and ZIKV [8], the high IgG positivity rates observed with both IgG ELISAs align with the known high seroprevalence of DENV in these regions [8,66]. The only slight decrease in positivity with the more stringent alternative cut-off using the Anti-DENV NS1 ELISA 2.0 (IgG), alongside consistently elevated antibody levels, suggest that the detected reactivity predominantly reflects true DENV exposure rather than non-specific cross-reactivity. Nevertheless, cross-reactivity with co-circulating flaviviruses or previous vaccination cannot be entirely excluded and remains a limitation when interpreting serological results in these regions.

Conversely, patients from mainland France, where flavivirus transmission is sporadic and rare [48], showed markedly lower IgG reactivity, with higher positivity in the Anti‑DENV NS1 ELISA 2.0 (IgG) than in the Anti-DENV Type 1–4 ELISA (IgG) (40% vs. 30%). Given the very small sample size (*n* = 10), and overlapping confidence intervals, this difference is likely attributable to sampling variability rather than a true performance difference.

These preliminary findings support the use of the Anti-DENV NS1 ELISA 2.0 (IgG) in diverse epidemiological contexts and the importance of cut-off adaptation where needed. Moreover, they highlight the risk of misclassification when serological findings are interpreted without clinical and epidemiological data, especially in areas with overlapping flavivirus circulation.

## Limitations

This study has several limitations. First, the relatively small number of patients with DENV, WNV, or ZIKV infection limits the statistical power of diagnostic accuracy estimates and restricts the generalizability of the findings. Second, dengue patients were exclusively recruited from Vietnam, and specificity cohorts originated from a limited number of settings, thereby restricting the geographical and epidemiological diversity of the study population. As only infections with DENV-1 and DENV-2, the predominant serotypes in Vietnam, were included, the results may not fully generalize to other serotypes. Third, the absence of classification into primary and secondary DENV infections limits the interpretation of NS1 antigenemia and antibody kinetics, as known differences between these infection types could not be systematically assessed. Fourth, although the blood donor panel originated from a non-endemic region (Northern Germany), prior flavivirus exposure could not be definitely excluded as vaccination and travel histories were unavailable. Fifth, the specificity of the NS1 and IgM ELISAs were not evaluated experimentally within the scope of this study due to resource constraints; instead, relevant data from previous publications were referenced. Sixth, longitudinal analyses were restricted by capping IgG values exceeding the assay’s upper limit of quantification, as systematic dilution testing for all such samples was outside the methodological scope of the study; nevertheless, overall kinetic trends remained clearly discernible. Finally, other commercial assays (ELISAs, rapid diagnostic tests etc.) were not included, limiting direct comparison of performance data. As this was a preliminary study, comprehensive multicenter studies based on larger and more diverse cohorts from different endemic settings, including all DENV serotypes and various detection methods, are warranted to further validate and extend our findings.

## Conclusion

The Euroimmun ELISAs provide a flexible and standardized framework for customized diagnostic strategies applicable to diverse clinical and epidemiological settings. By measuring serological markers with complementary diagnostic windows, these assays offer high diagnostic accuracy throughout all stages of DENV infection. In the acute phase, the combined use of NS1 antigen and IgM ELISAs detected all RT-PCR-positive dengue cases from single specimens, suggesting potential use in early diagnostics for laboratories without access to molecular diagnostics. The native antigen/gE-based IgG ELISA allows for early and sensitive IgG detection, though its specificity is limited. The new anti-DENV NS1 ELISA 2.0 (IgG), with its enhanced specificity and optional alternative cut-off, enables reliable differentiation of DENV-specific IgG from cross-reactive antibodies in regions where multiple flaviviruses co-circulate. This improved specificity is particularly relevant for epidemiological surveillance and serostatus determination. By specifically measuring the anti-NS1 IgG response, this assay captures DENV-specific IgG dynamics more accurately, which could provide an advantage in the use for convalescent-phase diagnostics. However, these findings should be regarded as preliminary and restricted to the studied settings and therefore require validation in larger multicenter studies involving more diverse cohorts from different endemic regions.

## Supporting information

S1 Fig Temporal dynamics of DENV NS1, anti-DENV IgM and anti-DENV IgG in 63 samples from dengue patients, grouped in days post onset (dpo). Samples were grouped into dpo intervals; the number of samples per interval is given in brackets below each group. No samples were available between 10 and 12 dpo. Data points represent mean values, and shaded areas around the lines represent standard deviation. Lines connecting data points are shown for visualization purposes and represent an assumed time course, not continuous measurements. For details on graphical elements (dashed lines, shaded areas, and thresholds), see Fig 1.
(PDF)

S2 Fig Individual time courses of DENV NS1, anti-DENV IgM and anti-DENV IgG levels in 22 dengue patients. Samples were collected at three distinct time points, except for patients #3, #4, and #11, for whom samples from only two time points were available in sufficient quantity for serological testing in this study. For IgG kinetics, the plots focus on the Anti-DENV NS1 ELISA 2.0 (IgG), given the study’s context within a seasonal dengue outbreak in a flavivirus-endemic region (Vietnam). Lines connecting discrete data points are shown for visualization purposes and represent an assumed time course, not continuous measurements. For details on graphical elements (dashed lines, shaded areas, and thresholds), see Fig 1.
(PDF)

S1 Table Characteristics of ELISAs used in this study. (DOCX)

S2 Table Qualitative ELISA results across three sampling time points (t1, t2, and t3) in 22 patients with PCR-confirmed DENV infection, sorted by days post onset (dpo). (DOCX)

S1 Dataset Anonymized dataset used for analysis. (XLSX)
