# Supplementary figures and images for: Improved and customized dengue serodiagnostics through combined NS1/IgM testing and novel dual-cut-off IgG ELISA
Source: PLoS Negl Trop Dis. 2026 Apr 27;20(4):e0014295. doi: 10.1371/journal.pntd.0014295 (PMC13152127; doi:10.1371/journal.pntd.0014295)

**DENV NS1 ELISA**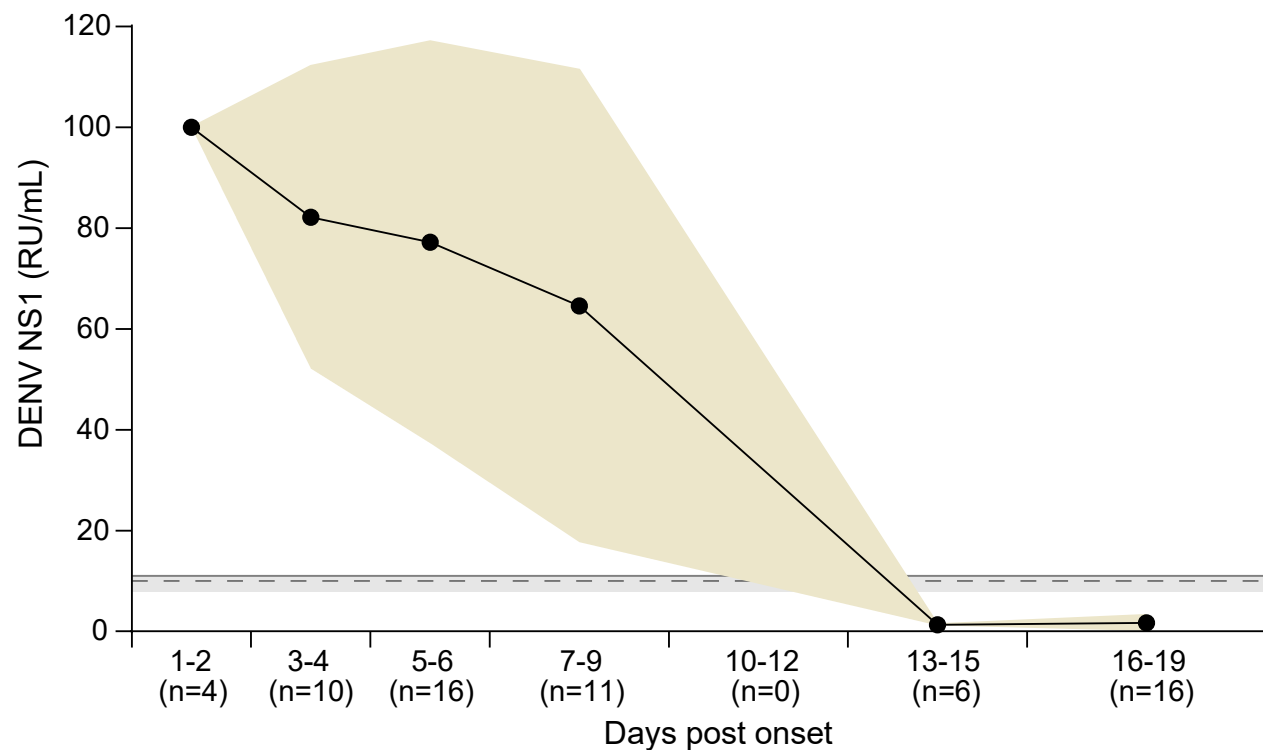**Anti-DENV Type 1-4 ELISA (IgM)**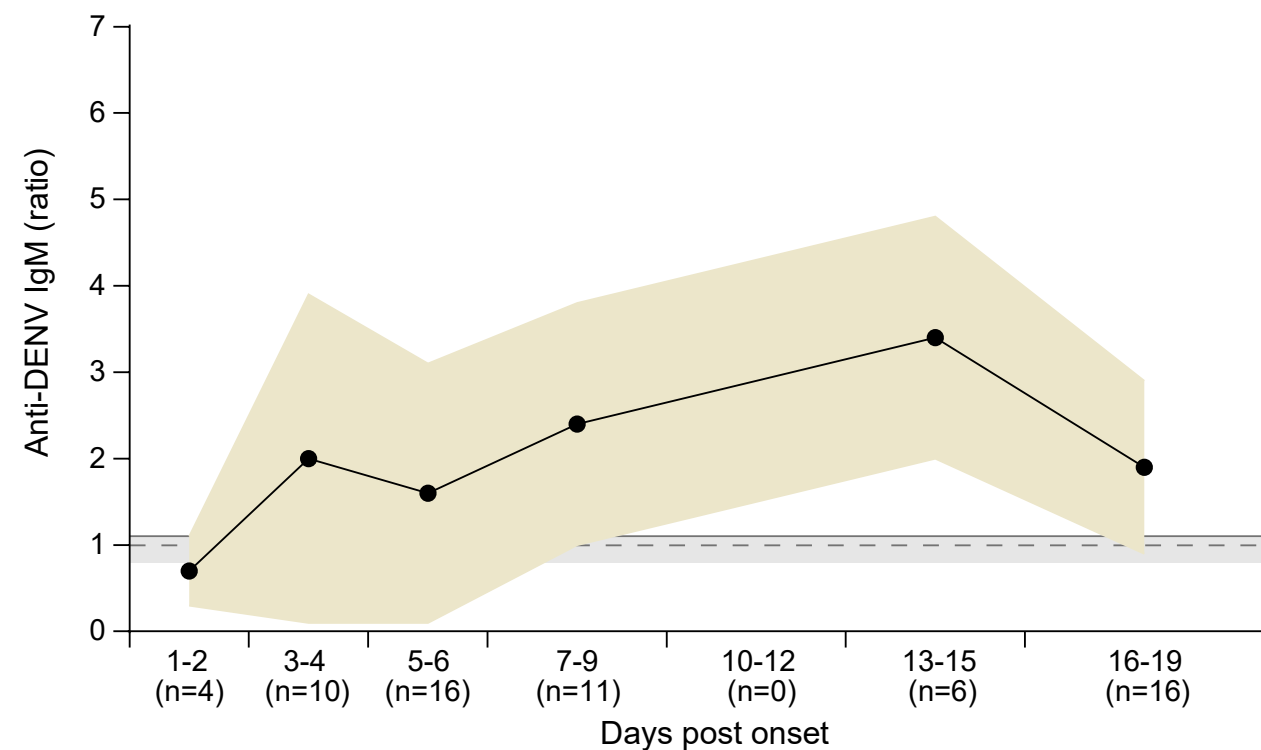**Anti-DENV Type 1-4 ELISA (IgG)**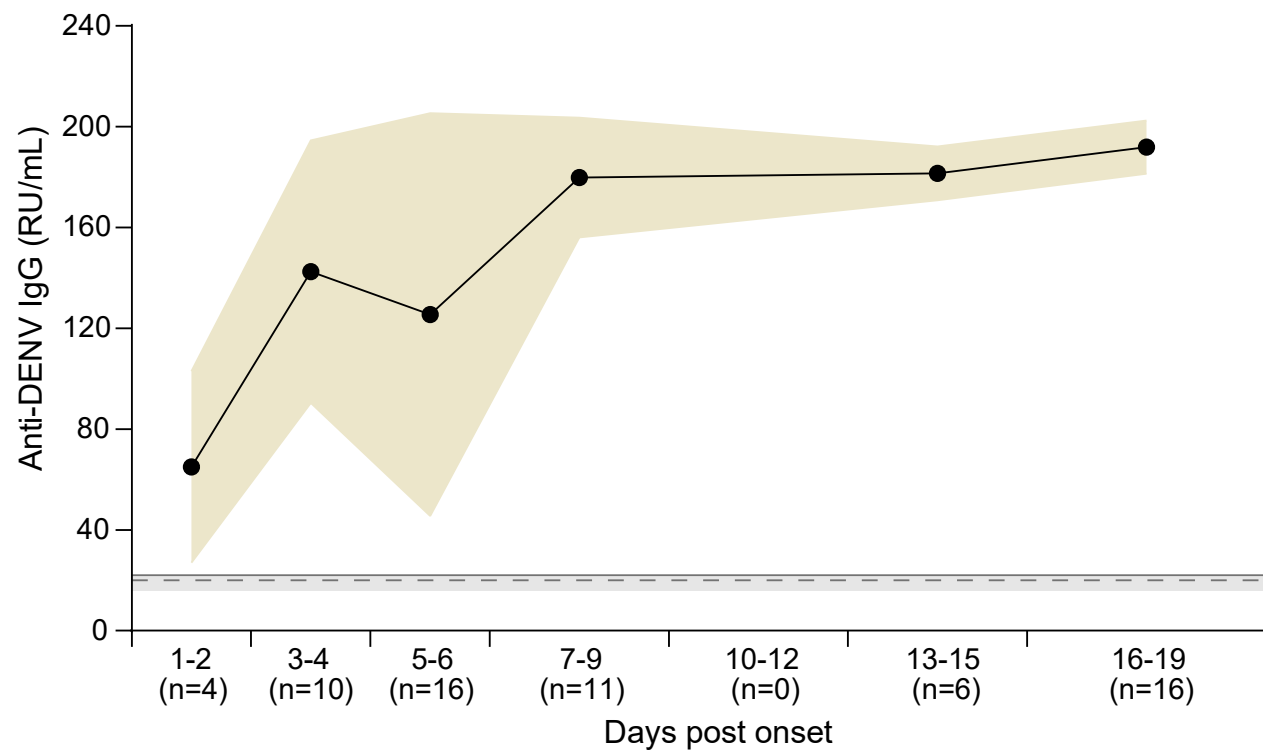**Anti-DENV NS1 ELISA 2.0 (IgG)**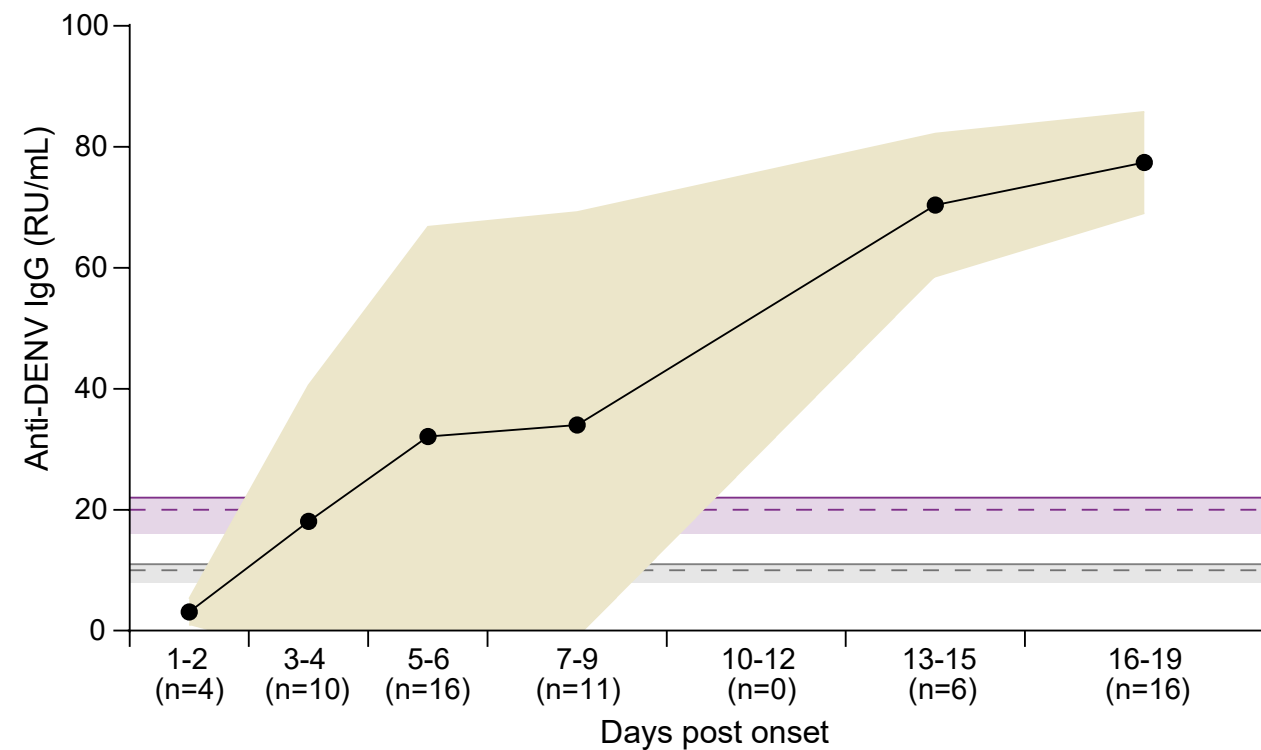

Supplement: S1 Fig — Samples were grouped into dpo intervals; the number of samples per interval is given in brackets below each group. No samples were available between 10 and 12 dpo. Data points represent mean values, and shaded areas around the lines represent standard deviation. Lines connecting data points are shown for visualization purposes and represent an assumed time course, not continuous measurements. For details on graphical elements (dashed lines, shaded areas, and thresholds), see Fig 1. (PDF) [file pntd.0014295.s001.pdf]

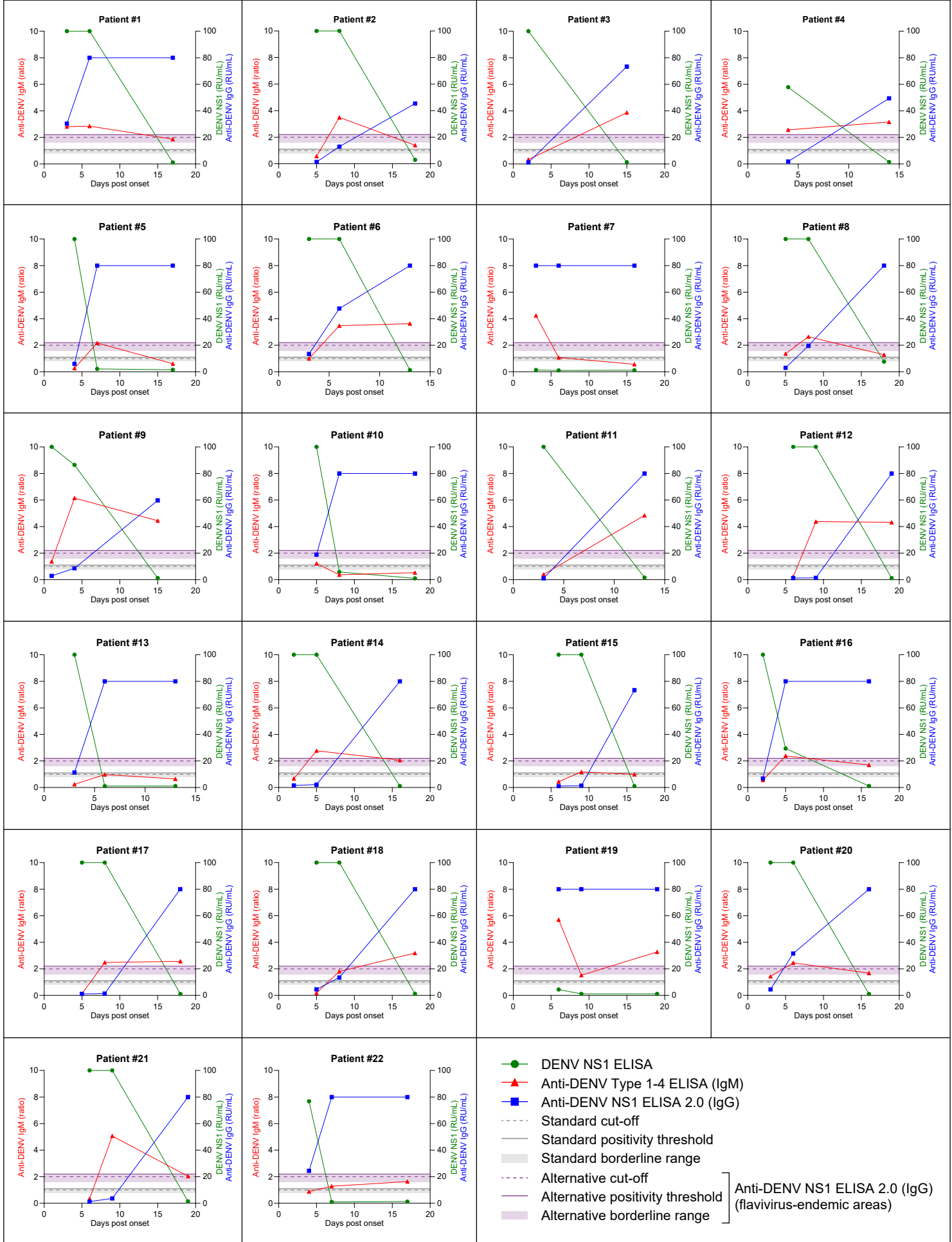

Supplement: S2 Fig — Samples were collected at three distinct time points, except for patients #3, #4, and #11, for whom samples from only two time points were available in sufficient quantity for serological testing in this study. For IgG kinetics, the plots focus on the Anti-DENV NS1 ELISA 2.0 (IgG), given the study’s context within a seasonal dengue outbreak in a flavivirus-endemic region (Vietnam). Lines connecting discrete data points are shown for visualization purposes and represent an assumed time course, not continuous measurements. For details on graphical elements (dashed lines, shaded areas, and thresholds), see Fig 1. (PDF) [file pntd.0014295.s002.pdf]
