# Supplementary material for: Improved and customized dengue serodiagnostics through combined NS1/IgM testing and novel dual-cut-off IgG ELISA
Source: PLoS Negl Trop Dis. 2026 Apr 27;20(4):e0014295. doi: 10.1371/journal.pntd.0014295 (PMC13152127; doi:10.1371/journal.pntd.0014295)
Supplement: S1 Table — (DOCX) [file pntd.0014295.s003.docx]

**S1 Table.** **Characteristics of ELISAs used in this study.**

|  | | **DENV NS1 ELISA** | **Anti-DENV Type 1-4 ELISA (IgM)** | **Anti-DENV Type 1-4 ELISA (IgG)** | **Anti-DENV NS1 ELISA 2.0 (IgG) ^a^** | |
| --- | --- | --- | --- | --- | --- | --- |
| **Manufacturer** | | Euroimmun | Euroimmun | Euroimmun | Euroimmun | |
| **Catalogue No.** | | EQ 266a-9601-1 | EI 266a-9601-1 M | EI 266a-9601-1 G | EI 266a-9601-3 G | |
| **Approval** | | CE-IVDD | CE-IVDD | CE-IVDD | CE-IVDR | |
| **System** | | Manual or automated | Manual or automated | Manual or automated | Manual or automated | |
| **Assay principle** | | Sandwich ELISA (antibody-coated microplate wells) | Indirect ELISA (antigen-coated microplate wells) | Indirect ELISA (antigen-coated microplate wells) | Indirect ELISA (antigen-coated microplate wells) | |
| **Format (total tests per kit)** | | 12 strips of 8 wells (96) | 12 strips of 8 wells (96) | 12 strips of 8 wells (96) | 12 strips of 8 wells (96) | |
| **Substrate** | | Monoclonal anti-dengue NS1 antibodies (mouse) | Highly purified virus particles and recombinant envelope gE of DENV1-4 | Highly purified virus particles and recombinant envelope gE of DENV1-4 | Recombinant NS1 of DENV1-4 | |
| **Analyte** | | NS1 | IgM | IgG | IgG | |
| **Serotype specificity** | | DENV1-4 | DENV1-4 | DENV1-4 | DENV1-4 | |
| **Sample type** | | Serum or plasma | Serum or plasma | Serum or plasma | Serum or plasma | |
| **Required test volume** | | 200 µL | 10 µL | 10 µL | 10 µL | |
| **Total incubation time** | | 135 min | 105 min | 105 min | 105 min | |
| **Semiquantitative evaluation ^b^** | |  |  |  |  | |
|  | **Ratio** | OD sample / OD calibrator 2 | OD sample / OD calibrator | OD sample / OD calibrator 2 | ― | |
|  | **Cut-off value** | OD calibrator 2 | OD calibrator | OD calibrator 2 | ― | |
|  | **Positive** | Ratio ≥1.1 | Ratio ≥1.1 | Ratio ≥1.1 | ― | |
|  | **Borderline** | Ratio ≥0.8 to <1.1 | Ratio ≥0.8 to <1.1 | Ratio ≥0.8 to <1.1 | ― | |
|  | **Negative** | Ratio <0.8 | Ratio <0.8 | Ratio <0.8 | ― | |
| **Quantitative evaluation ^b^** | |  |  |  |  | |
|  | **Standard curve (measurement range)** | 3 calibrators  (1/10/100 RU/mL) | ― | 3 calibrators  (2/20/200 RU/mL) | 5 calibrators  (1/10/20/40/80 RU/mL) | |
|  | **Cut-off value** | 10 RU/mL | ― | 20 RU/mL | Standard cut-off:  10 RU/mL | Alternative cut-off:  20 RU/mL ^c^ |
|  | **Positive** | ≥11 RU/mL | ― | ≥22 RU/mL | ≥11 RU/mL | ≥22 RU/mL |
|  | **Borderline** | ≥8 to <11 RU/mL | ― | ≥16 to <22 RU/mL | ≥8 to <11 RU/mL | ≥16 to <22 RU/mL |
|  | **Negative** | <8 RU/mL | ― | <16 RU/mL | <8 RU/mL | <16 RU/mL |

CE-IVDD, European conformity - in vitro diagnostic medical device directive; CE-IVDR, European conformity - in vitro diagnostic medical device regulation; DENV, dengue virus; DENV1-4, dengue virus serotypes 1 to 4; ELISA, enzyme-linked immunosorbent assay; gE, glycoprotein E; IgG, immunoglobulin G; IgM, immunoglobulin M; NS1, non-structural protein 1; OD, optical density.

^a^ Newly developed recombinant NS1-based anti-DENV IgG ELISA (version 2.0), compliant with IVDR, replacing the previous CE-IVDD-registered version (EI 266a-9601-2 G).

^b^ As recommended by the manufacturer.

^c^ An alternative cut-off can be applied to samples from flavivirus-endemic regions.
