# Supplementary material for: Improved and customized dengue serodiagnostics through combined NS1/IgM testing and novel dual-cut-off IgG ELISA
Source: PLoS Negl Trop Dis. 2026 Apr 27;20(4):e0014295. doi: 10.1371/journal.pntd.0014295 (PMC13152127; doi:10.1371/journal.pntd.0014295)
Supplement: S2 Table — (DOCX) [file pntd.0014295.s004.docx]

**S2 Table. Qualitative ELISA results across three sampling time points (t1, t2, and t3) in 22 patients with PCR-confirmed DENV infection, sorted by days post onset (dpo).**

| **Sampling time (dpo)** | **Patient No.** | **Sampling group** | **DENV**  **RT-PCR** | **DENV NS1 ELISA** | **Anti-DENV Type 1-4 ELISA (IgM)** | **Combined positivity**  **NS1/IgM ^a^** | **Anti-DENV**  **Type 1-4 ELISA (IgG)** | **Anti-DENV**  **NS1 ELISA 2.0 (IgG) ^b^** | |
| --- | --- | --- | --- | --- | --- | --- | --- | --- | --- |
|  |  |  |  |  |  |  |  | **Standard**  **cut-off** | **Alternative**  **cut-off** |
| 1 | 4 | t1 | pos | ND | | | | | |
| 1 | 9 | t1 | pos | pos | pos | + | neg | neg | neg |
| 2 | 3 | t1 | pos | pos | neg | + | pos | neg | neg |
| 2 | 14 | t1 | pos | pos | neg | + | pos | neg | neg |
| 2 | 16 | t1 | pos | pos | neg | + | pos | neg | neg |
| 3 | 1 | t1 | pos | pos | pos | + | pos | pos | pos |
| 3 | 6 | t1 | pos | pos | *bdl* | + | pos | pos | neg |
| 3 | 7 | t1 | pos | neg | pos | + | pos | pos | pos |
| 3 | 11 | t1 | pos | pos | neg | + | neg | neg | neg |
| 3 | 13 | t1 | pos | pos | neg | + | pos | pos | neg |
| 3 | 20 | t1 | pos | pos | pos | + | pos | neg | neg |
| 4 | 5 | t1 | pos | pos | neg | + | pos | neg | neg |
| 4 | 22 | t1 | pos | pos | *bdl* | + | pos | pos | pos |
| 4 | 4 | t2 | ND | pos | pos | + | pos | neg | neg |
| 4 | 9 | t2 | ND | pos | pos | + | pos | *bdl* | neg |
| 5 | 2 | t1 | pos | pos | neg | + | *bdl* | neg | neg |
| 5 | 8 | t1 | pos | pos | pos | + | pos | neg | neg |
| 5 | 10 | t1 | pos | pos | pos | + | pos | pos | *bdl* |
| 5 | 17 | t1 | pos | pos | neg | + | neg | neg | neg |
| 5 | 18 | t1 | pos | pos | neg | + | neg | neg | neg |
| 5 | 3 | t2 | ND | ND | | | | | |
| 5 | 14 | t2 | ND | pos | pos | + | pos | neg | neg |
| 5 | 16 | t2 | ND | pos | pos | + | pos | pos | pos |
| 6 | 12 | t1 | pos | pos | neg | + | neg | neg | neg |
| 6 | 15 | t1 | pos | pos | neg | + | pos | neg | neg |
| 6 | 19 | t1 | pos | neg | pos | + | pos | pos | pos |
| 6 | 21 | t1 | pos | pos | neg | + | neg | neg | neg |
| 6 | 1 | t2 | ND | pos | pos | + | pos | pos | pos |
| 6 | 6 | t2 | ND | pos | pos | + | pos | pos | pos |
| 6 | 7 | t2 | ND | neg | *bdl* | - | pos | pos | pos |
| 6 | 11 | t2 | ND | ND | | | | | |
| 6 | 13 | t2 | ND | neg | *bdl* | - | pos | pos | pos |
| 6 | 20 | t2 | ND | pos | pos | + | pos | pos | pos |
| 7 | 5 | t2 | ND | neg | pos | + | pos | pos | pos |
| 7 | 22 | t2 | ND | neg | pos | + | pos | pos | pos |
| 8 | 2 | t2 | ND | pos | pos | + | pos | pos | neg |
| 8 | 8 | t2 | ND | pos | pos | + | pos | pos | *bdl* |
| 8 | 10 | t2 | ND | neg | neg | - | pos | pos | pos |
| 8 | 17 | t2 | ND | pos | pos | + | pos | neg | neg |
| 8 | 18 | t2 | ND | pos | pos | + | pos | pos | neg |
| 9 | 12 | t2 | ND | pos | pos | + | pos | neg | neg |
| 9 | 15 | t2 | ND | pos | pos | + | pos | neg | neg |
| 9 | 19 | t2 | ND | neg | pos | + | pos | pos | pos |
| 9 | 21 | t2 | ND | pos | pos | + | pos | neg | neg |
| 13 | 6 | t3 | ND | neg | pos | + | pos | pos | pos |
| 13 | 11 | t3 | ND | neg | pos | + | pos | pos | pos |
| 13 | 13 | t3 | ND | neg | neg | - | pos | pos | pos |
| 14 | 4 | t3 | ND | neg | pos | + | pos | pos | pos |
| 15 | 3 | t3 | ND | neg | pos | + | pos | pos | pos |
| 15 | 9 | t3 | ND | neg | pos | + | pos | pos | pos |
| 16 | 7 | t3 | ND | neg | neg | - | pos | pos | pos |
| 16 | 14 | t3 | ND | neg | pos | + | pos | pos | pos |
| 16 | 15 | t3 | ND | neg | *bdl* | - | pos | pos | pos |
| 16 | 16 | t3 | ND | neg | pos | + | pos | pos | pos |
| 16 | 20 | t3 | ND | neg | pos | + | pos | pos | pos |
| 17 | 1 | t3 | ND | neg | pos | + | pos | pos | pos |
| 17 | 5 | t3 | ND | neg | neg | - | pos | pos | pos |
| 17 | 22 | t3 | ND | neg | pos | + | pos | pos | pos |
| 18 | 2 | t3 | ND | neg | pos | + | pos | pos | pos |
| 18 | 8 | t3 | ND | neg | pos | + | pos | pos | pos |
| 18 | 10 | t3 | ND | neg | neg | - | pos | pos | pos |
| 18 | 17 | t3 | ND | neg | pos | + | pos | pos | pos |
| 18 | 18 | t3 | ND | neg | pos | + | pos | pos | pos |
| 19 | 12 | t3 | ND | neg | pos | + | pos | pos | pos |
| 19 | 19 | t3 | ND | neg | pos | + | pos | pos | pos |
| 19 | 21 | t3 | ND | neg | pos | + | pos | pos | pos |

bdl, borderline; DENV, dengue virus; dpo, days post onset; ELISA, enzyme-linked immunosorbent assay; IgG, immunoglobulin G; IgM, immunoglobulin M; ND, not determined at this time point; neg, negative; NS1, non-structural protein 1; pos, positive; RT-PCR, real-time reverse transcription polymerase chain reaction.

^a^ Combined positivity (‘+’) was defined as positive reactivity in at least one of the two ELISAs (DENV NS1 or anti-DENV IgM).

^b^ An alternative cut-off (20 RU/mL) may be applied for samples from flavivirus-endemic areas, instead of the standard cut-off (10 RU/mL).
